# An Integrated Genetic and Cytogenetic Map for Zhikong Scallop, *Chlamys farreri*, Based on Microsatellite Markers

**DOI:** 10.1371/journal.pone.0092567

**Published:** 2014-04-04

**Authors:** Liying Feng, Liping Hu, Xiaoteng Fu, Huan Liao, Xuan Li, Aibin Zhan, Lingling Zhang, Shi Wang, Xiaoting Huang, Zhenmin Bao

**Affiliations:** 1 Key Laboratory of Marine Genetics and Breeding (MGB), Ministry of Education, College of Marine Life Sciences, Ocean University of China, Qingdao, China; 2 Yantai Fisheries Research Institute, Yantai, China; 3 Research Center for Eco-Environmental Sciences, Chinese Academy of Sciences, Beijing, China; George Washington University School of Medicine and Health Sciences, United States of America

## Abstract

The reliability of genome analysis and proficiency of genetic manipulation requires knowledge of the correspondence between the genetic and cytogenetic maps. In the present study, we integrated cytogenetic and microsatellite-based linkage maps for Zhikong scallop, *Chlamys farreri*. Thirty-eight marker-anchored BAC clones standing for the 19 linkage groups were used to be FISH probes. Of 38 BAC clones, 30 were successfully located on single chromosome by FISH and used to integrate the genetic and cytogenetic map. Among the 19 linkage groups, 12 linkage groups were physically anchored by 2 markers, 6 linkage groups were anchored by 1 marker, and one linkage group was not anchored any makers by FISH. In addition, using two-color FISH, six linkage groups were distinguished by different chromosomal location; linkage groups LG6 and LG16 were placed on chromosome 10, LG8 and LG18 on chromosome 14. As a result, 18 of 19 linkage groups were localized to 17 pairs of chromosomes of *C. farreri*. We first integrated genetic and cytogenetic map for *C. farreri*. These 30 chromosome specific BAC clones in the cytogenetic map could be used to identify chromosomes of *C. farreri*. The integrated map will greatly facilitate molecular genetic studies that will be helpful for breeding applications in *C. farreri* and the upcoming genome projects of this species.

## Introduction

Zhikong scallop, *Chlamys farreri* Jones et Preston 1904, is naturally distributed around the sea coasts of North China, Korea and Japan [1]. It is a species of great economic importance in China. The production has reached approximately 80% of the shellfish aquaculture production in China [2]. According to the statistic data in China fishery statistical yearbook, the annual production of scallop has exceeded 1,300,000 tons in 2011 [3]. With the rapid development of aquaculture in about ten years, the genetic structure of the *C. farreri* populations has been affected and the genetic diversity of the selected population has a descendent trend [4]–[6]. Some problems including declining production and disease outbreaks bring new challenge for breeding science [7]–[9]. The efficient breeding program depends on the availability of genetic resources such as saturated genetic linkage maps. Several linkage maps, such as AFLP linkage maps [10]–[12] and microsatellite-based linkage maps [13], have been constructed and some QTL associated with economic traits including shell length, shell width, shell height and gross weight have been identified [13]. These linkage maps and QTLs provided useful information for marker-assisted selection of *C. farreri*. Recently, some genomic information of *C. farreri* has been obtained through the high-throughput sequencing technology, such as transcriptomic sequences (NCBI Short Read Archive (SRA) database, SRA030509) and genome sequences (SRP018107). The genetic map and genomic information will provide the powerful resources for the upcoming genome project about *C. farreri*. A key process in the genome assembly is to determine and verify the precise physical location and order of the large sequence blocks (scaffolds). Nevertheless, linkage maps are based on recombination rates which always do not occur uniformly along a chromosome. Linkage maps could show the right order of markers, but it couldn't provide their precise physical distance. As a result, the completeness of a genome map is difficult to assess only based on the genetic map. A cytogenetic map could assign linkage group with specific chromosome and visibly integrate the genetic recombination rates with physical distances along each chromosome. However, the chromosomal identification is still infeasible for *C. farreri* and the relationship between the linkage groups and their corresponding chromosomes is still unknown [14].

Fluorescence in situ hybridization (FISH) is a powerful tool to define the cytogenetic location and relative order of DNA sequences, thereby anchoring the genome sequence to the chromosomes. It has been successfully used to integrate genetic and cytogenetic maps in many plants and animals [15]–[18]. In Pecitinidae, FISH technology has been proved to be an effective method and widely used in chromosomal investigation, such as chromosome identification [14], gene mapping [19]–[29] and chromosome rearrangements [30], [31]. *C. farreri*, a member of family Pectinidae, has a haploid number of 19 with a karyotype of 3 m+5 sm+11 st. The number of chromosomal arms is 38 which is the highest number of chromosomal arms in Pectinidae. As a result, *C. farreri* is considered as the closest representative of the ancestral karyotype of Pectinidae [30], [32]. With the application of FISH in recent years, some cytogenetic researches have been carried out for *C. farreri*. Repetitive genes including major and minor rRNA have been located on one pair of subtelomeric chromosome [23], [30]. Histone H3 gene were located on short arm of a large submetacentric chromosome [31]. 8 fosmid clones have been used to identify 8 chromosomes of *C. farreri* [14]. However, these chromosomes were not associated with linkage groups.

In this study, to integrate the genetic and cytogenetic maps, we selected markers from microsatellite-based linkage maps to screen *C. farreri* BAC libraries. These anchored BAC clones were labeled as FISH probes to hybridize to chromosomes of *C. farreri*. As a result, the relationship between chromosomes and the linkage groups (LGs) in *C. farreri* has been established. The construction of an integrated map should greatly facilitate molecular genetic studies and the upcoming genome sequence projects of *C. farreri*.

## Materials and Methods

### Selection of marker-anchored BACs and Probe Labeling

In previous study, the microsatellite-based linkage map of *C. farreri* was constructed, which contained 19 linkage groups, 154 markers and spanned 1561.8 cM with an average inter marker spacing of 12.3 cM and 77.0% genome coverage [13]. Forty-two markers from microsatellite-based linkage maps of *C. farreri* were selected for the purpose of finding marker-anchored BACs. Each of the 19 linkage groups was represented by at least two markers. The marker-anchored BACs were screened by 4D, two-step PCR [14]. Briefly, the first step was performed by PCR using primer sets for selected microsatellite markers and using superpools as a template to determine which superpools contained the markers. Then, in the second step, 4D-PCR with the primer sets was performed on the clones of the superpools containing the markers. The condition of PCR was according to the reference no. 13 and the primer sequences for microsatellites were shown in Table S1. The *Hind*III-BAC (BH) and *Bam*HI-BAC (BB) libraries of *C. farreri* [33] used to isolate the microsatellite markers were constructed from the adductor muscle, mantle and gill of one adult scallop and based on two restriction enzymes, *Hind*III and *Bam*HI respectively. Screened 38 positive BAC clones were confirmed further by sequence analysis of PCR products. A routine phenol/chloroform extraction method was used to extract and purify the BAC genomic DNA. The BAC DNAs were then labeled with digoxigenin (Dig)-11-dUTP or biotin-16-dUTP by Dig- or Biotin-nick translation mix (Roche) according to the manufacturer's instructions.

### Chromosome Preparation

The trochophore larvae were obtained according to Huang et al. [23]. The collected scallop larvae were treated with colchicine (0.01%) for 2 h at room temperature, and then exposed to 0.075 M KCl solution for 20 min, finally, fixed three times (15 min each) in the fresh Carnoy's solution (100% ethanol: glacial acetic acid, 3∶1). The fixed larvae were dissociated into a cell suspension using 50% acetic acid, and then dropped onto hot-wet slides, air-dried and stored at −20°C until required.

### Preparation of C_0_t-1 DNA

Genomic DNA from *C. farreri* adductor muscle was extracted by standard phenol-chloroform methods [34]. Then, *C_0_t*-1 DNA representing moderately to highly repetitive sequences has been prepared according to Hu et al. [35] and used as hybridization inhibitors.

### Fluorescence in situ hybridization

Chromosome slides were pretreated with RNase A (100 µg/mL) in 2×SSC at 37°C for 1 h, followed by pepsin (0.005%) in 10 mM HCl at 37°C for 10 min. Chromosome slides were denatured in 70% formamide mixed with 2×SSC at 75°C for 2 min, immediately dehydrated in a chilled ethanol series (70%, 90% and 100%) for 5 min each, and then air-dried. The probe hybridization mixture consisted of 10–15 ng/µl Dig-11-dUTP and/or biotin-16-dUTP labeled BAC DNAs, 50% deionized formamide, 10% dextran sulfate, 2×SSC. When necessary, *C. farreri C_0_t-1* DNA (20 ng/µl) was also added to the hybridization mixture. The probe mixture was denatured at 80°C for 5 min and cooled immediately. Subsequently, the hybridization mixture was applied to chromosome preparations, and the slides were incubated in a moist chamber at 37°C for 16–18 h. Next, slides were washed in 50% formamide in 2×SSC at 37°C for 10 min, in 1×SSC at 37°C three times (5 min each), and in 2×SSC at room temperature for 5 min. Dig-labeled and biotin-labeled probes were detected using anti-digoxigenin-rhodamine (Roche) and fluorescein avidin DCS (Vector), respectively. Chromosomes were then counterstained with 4, 6-diamidino-2-phenylindole (DAPI) or propidium iodide (PI) (Vector). For two-color FISH, probes labeled with Dig and biotin were pooled for hybridization and detected on DAPI counterstained chromosomes.

Images were captured by a CCD camera attached to a Nikon Eclipse-600 epifluorescence microscope. Grey-scale images on each color channel were captured, pseudo-colored and merged by Lucia-FISH Image System. Karyotype analysis was carried out according to criteria defined by Levan et al. [36].

## Results

### Isolation of marker-anchored BACs

Forty-two markers from the microsatellite-based linkage map were used to screen by 4D, two-step PCR from *C. farreri* BAC libraries (Table S1). 38 marker-anchored BAC clones were isolated and confirmed by sequencing (Table 1). These 38 BAC clones were then used to integrate the genetic map and cytogenetic map by FISH.

**Table 1:** Chromosomal location description of the SSR-BAC clones.

Linkage Group	Locus name	BAC code	Location of signals	*C_0_t*-1 DNA used
LG1	CFLD006	BH1304E11	On single chromosome pair (8q)	No
LG1	CFFD143	BH1162H2	On single chromosome pair (8q)	Yes
LG2	CFBD213	BH783B4	On single chromosome pair (12q)	Yes
LG2	CFKD077	BH793B11	On single chromosome pair (12p)	Yes
LG3	CFHD004	BH799B12	On single chromosome pair (7p)	Yes
LG3	CFFD093	BH1049A9	On single chromosome pair (7q)	No
LG4	CFFD048	BB311A9	On single chromosome pair (2q)	Yes
LG4	CFAD021	BB322D4	On centromeric region of multiple chromosomes	Yes
LG5	CFFD144	BB105A1	On single chromosome pair (18p)	Yes
LG5	CFAD018	BB86D4	On single chromosome pair (18q)	Yes
LG6	CFBD170	BH1285H8	On single chromosome pair (10p)	Yes
LG6	CFCD104	BB24H11	On centromeric region of multiple chromosomes	Yes
LG7	CFFD110	BH1291D12	On single chromosome pair (13q)	No
LG7	CFLD034	BH1060D10	No signals	Yes
LG8	CFCD172	BH966F2	On single chromosome pair (14q)	Yes
LG8	CFLD047	BB138G4	On centromeric region of multiple chromosomes	Yes
LG9	CFFD147	BB224E11	On 3 pairs of chromosomes	Yes
LG9	CFFD061	BB98C5	On 2 pairs of chromosomes	Yes
LG10	CFJD077	BB39F11	On single chromosome pair (19q)	Yes
LG10	CFFD167	BB27B5	Scattered across the chromosomes	Yes
LG11	CFBD204	BB75B6	On single chromosome pair (15p)	Yes
LG11	CFBD193	BH1308E3	On single chromosome pair (15q)	Yes
LG12	CFKD091	BH885H2	On single chromosome pair (11p)	Yes
LG12	CFKD096	BB224B4	On single chromosome pair (11p)	Yes
LG13	CFOD062	BB233G7	On single chromosome pair (6q)	Yes
LG13	CFKD022	BB312B11	On single chromosome pair (13p)	Yes
LG14	CFMSP003	BH984B5	On single chromosome pair (1q)	Yes
LG14	CFJD047	BB105B2	On single chromosome pair (1q)	Yes
LG15	CFID005	BB235A11	On single chromosome pair (19q)	Yes
LG15	CFBD169	BH377G2	On centromeric region of multiple chromosomes	Yes
LG16	CFMSM014	BH565D6	On single chromosome pair (4q)	Yes
LG16	CFFD041	BB69B10	On single chromosome pair (10p)	Yes
LG17	CFLD144	BH368F12	On single chromosome pair (16q)	Yes
LG17	CFOD056	BH1261C3	On single chromosome pair (16q)	Yes
LG18	CFBD224	BH1003G1	On single chromosome pair (11q)	No
LG18	CFAD184	BH986B2	On single chromosome pair (14q)	Yes
LG19	CFE15	BH431C4	On single chromosome pair (9q)	Yes
LG19	CFLD060	BB239A6	On single chromosome pair (17q)	Yes

### BAC-FISH signal strength and distribution

**Figure 1:**
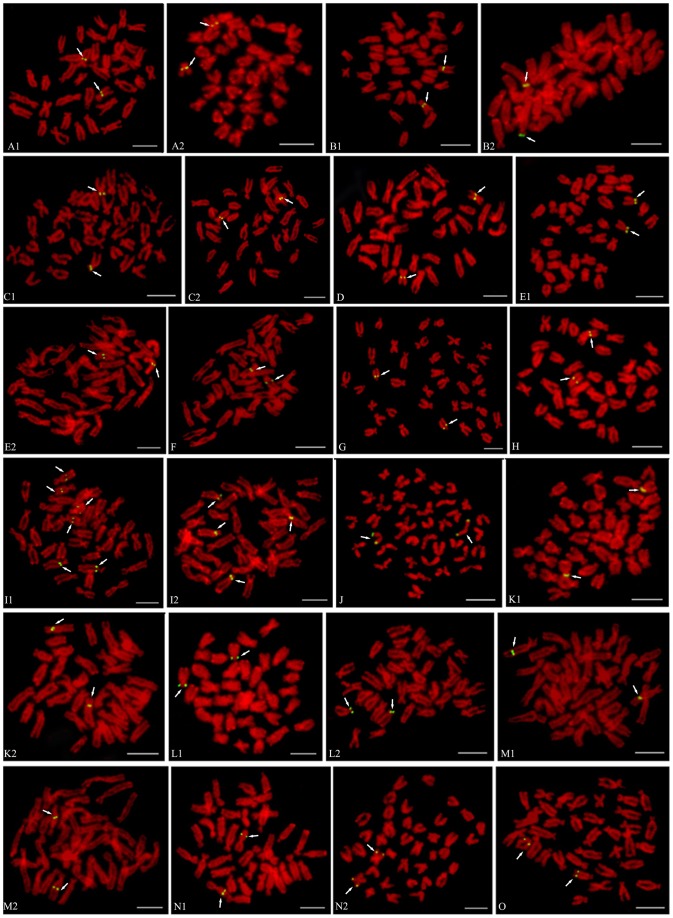
FISH results of 24 BAC clones from LG1 to LG15 with specific signal on *C. farreri* chromosomes. (A1) BH1304, (A2) BH1162H2, (B1) BH783B4, (B2) BH793B11, (C1) BH799B12, (C2) BH1049A9, (D) BB311A9, (E1) BB105A1, (E2) BB86D4, (F) BH1285H8, (G) BH1291D12, (H) BH966F2, (I1) BB224E11, (I2) BB98C5, (J) BB39F11, (K1) BB75B6, (K2) BH1308E3, (L1) BH885H2, (L2) BB224B4, (M1) BB233G7, (M2) BB312B11, (N1) BH984B5, (N2) BB105B2, (O) BB235A11. Bars = 5 µm.

**Figure 2:**
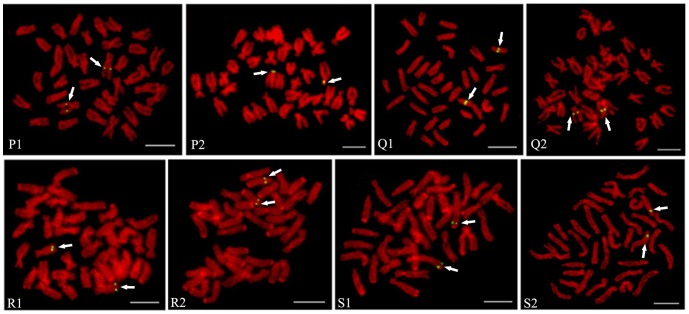
FISH results of 8 BAC clones from LG16, LG17 and LG18 with specific signal on *C. farreri* chromosomes. (P1) BH565D6, (P2) BB69B10, (Q1) BH368F12, (Q2) BH1261C3, (R1) BH1003G1, (R2) BH986B2, (S1) BH431C4, (S2) BB239A6. Bars = 5 µm.

**Figure 3:**
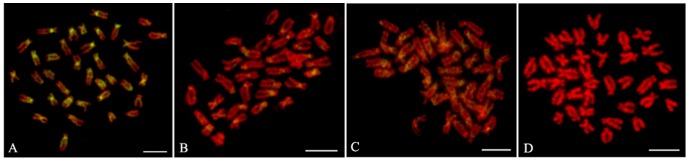
FISH results of microsatellite-anchored BAC clones without specific signal on chromosomes. (A) BB322D4 without *C_0_t*-1 DNA blocking, (B) BB322D4 with *C_0_t*-1 DNA blocking, (C) BB27B5 with *C_0_t*-1 DNA blocking, (D) BH1060D10 with *C_0_t*-1 DNA blocking. Bars = 5 µm.

Many BACs required the inclusion of *C_0_t*-1 DNA in the probe mixture. Because *C_0_t*-1 DNA blocked the hybridization of the repetitive DNA in the BAC clones, signals from nonspecific hybridization sites were reduced and the signal from the specific hybridization site of the probe could be distinguished more easily. Of the 38 BAC clones placed on the cytogenetic maps, only four (BH1304E11, BH1049A9, BH1291D12 and BH1003G1) gave clear signals without *C_0_t*-1 DNA (Figure 1, 2), twenty-six BAC clones produced specific FISH signals on one pair of chromosomes with the aid of *C_0_t*-1 DNA (Figure 1, 2), two BAC clones hybridized to multiple chromosomes stably (Figure 1 I1, I2). There were another 6 BAC clones which didn't produce specific hybridization sites even with *C_0_t*-1 DNA (Figure 3). Four of these, hybridized strongly to the centromeric region of multiple chromosomes, although the signals obtained with these BAC clones were significantly reduced with increasing amounts of *C_0_t*-1 DNA, the specific hybridization site could not be identified (Figure 3 A, B). The signals produced by BAC clone, BB27B5, were scattered across the chromosomes, but all these signals were reduced when *C_0_t*-1 DNA was used, suggesting that a large proportion of this BAC clone consisted of repetitive DNA (Figure 3 C). Only one BAC clone, BH1060D10, didn't show any signal on the chromosomes of *C. farreri* (Figure 3 D). As a result, we finally used those 30 BAC clones produced specific FISH signals on one pair of chromosomes to integrate the genetic and cytogenetic map.

### Assignment of linkage groups to chromosomes

**Figure 4:**
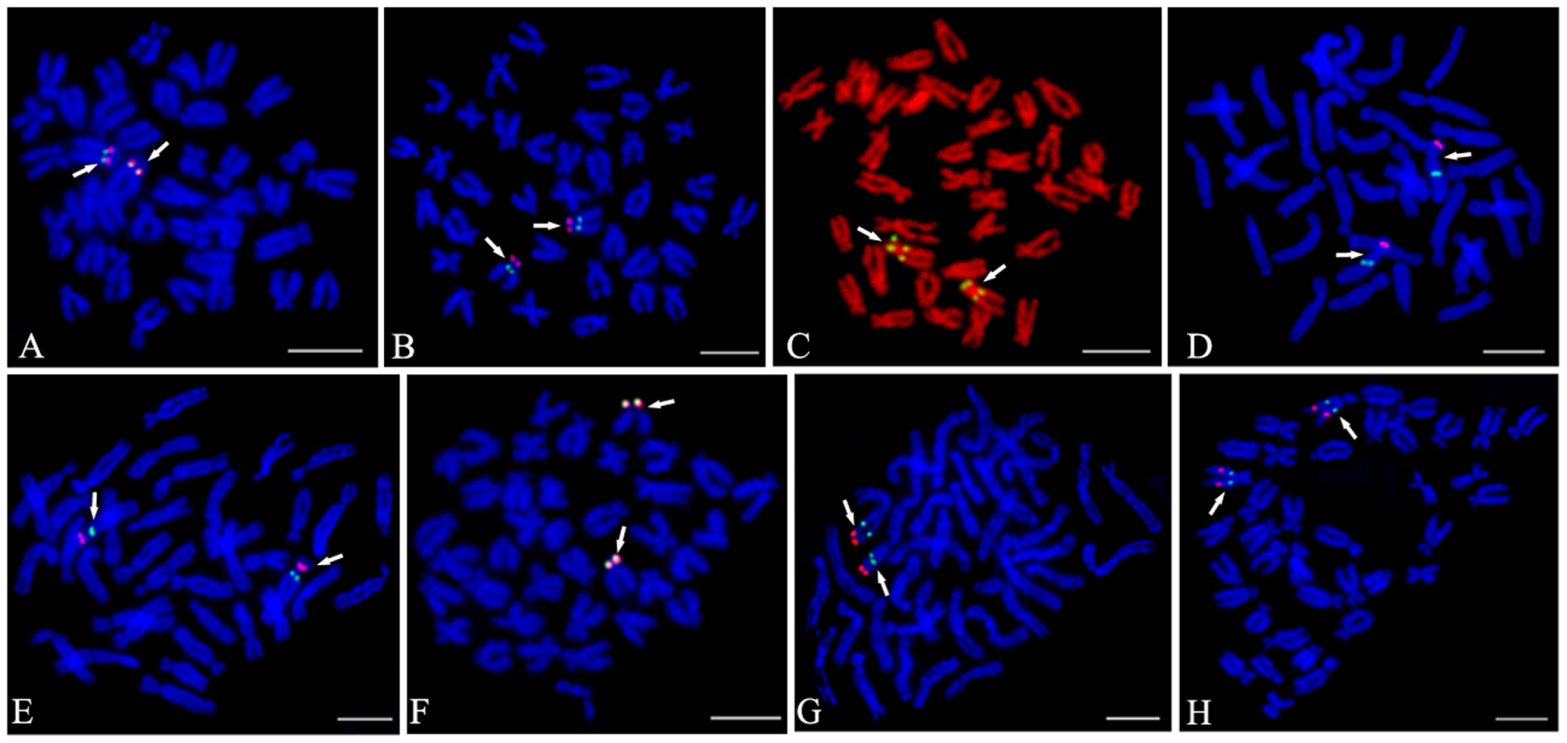
Two-color FISH results of the different BAC clones. (A) LG1, BH1304E11 (Red) and BH1162H2 (Green);(B) LG2, BH783B4 (Green) and BH793B11 (Red); (C) LG3, BH799B12 (Green) and BH1049A9 (Green);(D) LG5, BB105A1 (Red) and BB86D4 (Green);(E) LG11, BB75B6 (Red) and BH1308E3 (Green);(F) LG12, BH885H2 (Red) and BB224B4 (Green);(G) LG14, BH984B5 (Green) and BB105B2 (Red);(H) LG17, BH368F12 (Red) and BH1261C3 (Green). Bars = 5 µm.

**Figure 5:**
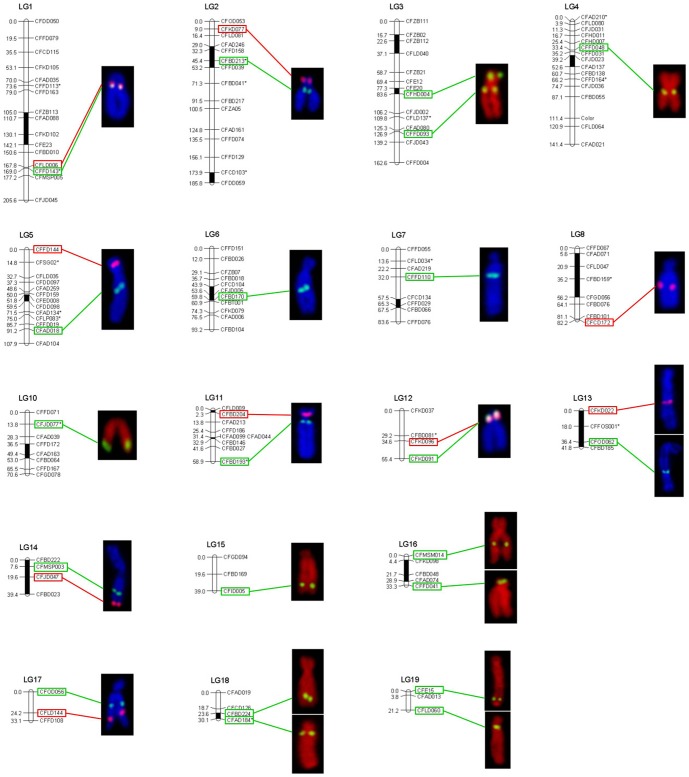
Integration of the 18 genetic linkage groups with individual chromosomes of Zhikong scallop, *C. farreri*.

FISH analysis was used to integrate the linkage groups to the chromosomes of *C. farreri*. Among the 19 linkage groups, 12 linkage groups were physically anchored by 2 markers, 6 linkage groups were anchored by 1 marker, and one linkage group (LG9) was not anchored any makers by FISH (Table 1). After co-hybridization, the 2 BAC clones from 8 linkage groups (LG1, LG2, LG3, LG5, LG11, LG12, LG14, and LG17) were hybridized to the corresponding one pair of chromosomes (Figure 4 A–H). However, the two BAC clones from four linkage groups (LG13, LG16, LG18, and LG19) were hybridized on different chromosomes. The BAC clones (BB233G7 and BB312B11) from LG13 were hybridized on chromosome 6 and 13 separately by co-hybridization (Figure S3). The co-hybridization signals of 2 BAC clones from LG16, LG18, and LG19 were weakened seriously and the relationship between these clones were obtained only by karyotyping analysis. Two BAC clones (BH565D6 and BH69B10) from LG16 were assigned to chromosome 4 and 10, 2 BAC clones (BH986B2 and BH1003G1) from LG18 on chromosome 11 and 14, 2 BAC clones (BB239A6 and BH431C4) from LG19 on chromosome 9 and 17 (Figure S4). As a result, using the 30 BAC clones, 18 of 19 linkage groups (except LG9) were assigned to 17 pairs of chromosomes of *C. farreri* (Figure 5). Furthermore, the 30 chromosome specific BAC clones could be used as cytological markers in the future cytogenetic studies of *C. farreri*.

**Figure 6:**
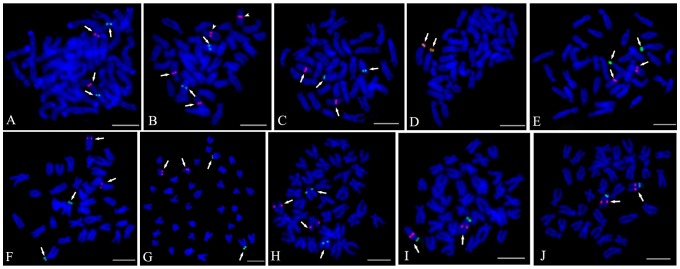
Two-color FISH results of the different linkage groups. (A) LG13, Chromosome 6, BB233G7 (Red) and Chromosome 6, F458F11 (Green); (B) LG1, Chromosome 8, BH1304E11 (Red, arrow head), LG3, Chromosome 7, BH1049A9 (Red, arrow) and Chromosome 6, F458F11 (Green); (C) LG3, Chromosome 7, BH1049A9 (Red) and LG18, Chromosome 11, BH1003G1 (Green); (D) LG16, Chromosome 10, BB69B10 (Red) and Chromosome 10, F408A12 (Green); (E) LG1, Chromosome 8, BH1304E11(Red) and Chromosome 10, F408A12 (Green); (F) LG18, Chromosome 11, BH1003G1 (Red) and Chromosome 10, F408A12 (Green); (G) LG7, Chromosome 13, BH1291D12 (Red) and Chromosome 10, F408A12 (Green); (H) LG7, Chromosome 13, BH1291D12 (Green) and LG18, Chromosome 11, BH1003G1 (Red); (I) LG6, BH1285H8 (Red) and LG16 BB69B10 (Green); (J) LG8, BH966F2 (Red) and LG18, BH986B2 (Green). Bars = 5 µm.

In order to verify different linkage groups are located on different chromosomes, we conducted karyotype analysis (Figure S1, S2, S3 and S4) and two-color FISH (Figure 6). *C. farreri* possesses 3 pairs of metacentric chromosome, 5 pairs of submetacentric chromosome and 11 pairs of subtelocentric chromosome. The 3 pairs of metacentric chromosome could be easily distinguished from the other 16 pairs of chromosome directly by karyotype analysis because of the apparent morphological difference [23]. Therefore, the LG14 and LG4 which were assigned to metacentric chromosome 1 and 2 could be easily distinguished by karyotype analysis (Figure S1-LG4, Figure S3-LG14). As for the other linkage groups which were assigned to the submetacentric or subtelocentric chromosomes, two-color FISH was first applied using the four BAC clones that could be successfully mapped without *C_0_t*-1 DNA. Then other two fosmid clones (F458F11and F408A12) reported in previous study were also used for two-color FISH [14]. F458F11 was localized on one pair of submetacentric chromosome (Figure 6 A) and F408A12 was localized on one pair of subtelocentric chromosome (Figure 6 D). Based on two-color FISH, LG1 (Chromosome 8), LG3 (Chromosome 7) and LG13 (Chromosome 6) were confirmed to be assigned to different chromosomes (Figure 6 A, B). So were LG3 (Chromosome 7) and LG18 (Chromosome 11) (Figure 6 C), LG1 (Chromosome 8) and LG16 (Chromosome 10) (Figure 6 D, E), LG18 (Chromosome 11) and LG16 (Chromosome 10) (Figure 6 D, F), LG7 (Chromosome 13) and LG16 (Chromosome 10) (Figure 6 D, G), as well as LG18 (Chromosome 11) and LG7 (Chromosome 13) (Figure 6 H). In addition, two linkage groups LG6 and LG16 were placed on the same chromosome 10 by the co-hybridization of 2 BAC clones (BH1285H8 and BB69B10) (Figure 6 I), LG8 and LG18 were connected by the co-hybridization of 2 BAC clones (BH966F2 and BH986B2) on chromosome 14 (Figure 6 J).

### Orientation of linkage groups with chromosomes

According to the FISH signal positions of BAC clones, the orientation of all 19 *C. farreri* linkage groups has been evaluated. The genetic and the physical position of the marker in 3 linkage groups, including LG1, LG3, LG10, are in opposite orientation, therefore, these 3 linkage groups has to be reorientated the ends of the short arm to the north side of the linkage maps and the long arm to the south side.

## Discussion

FISH has been proved to be an efficient method to correlate genetic and cytogenetic maps by using marker-anchored BAC clones. It has been widely used in mapping eukaryotic genome [15], [17], [37], [38]. Since the BAC libraries are composed of genomic clones that are 100–200 kb in size, and the targets of FISH are thus relatively large and easy to detect. However, some BAC clones contain dispersed repetitive sequences that cause high levels of FISH background signal. *C_0_t*-1 DNA was always used to block the repetitive DNA. In *Brassica oleracea*, 4 of 17 BAC clones gave clear signals without *C_0_t*-1 DNA and 11 BAC clones produced specific hybridization site with *C_0_t*-1 DNA, finally, 9 linkage groups were assigned to 9 chromosomes and the orientation of 4 linkage groups was revised [39]. In duck, *Anas platyrhynchos*, 24 of 28 BAC clones were detected definitely on chromosomes and 11 of 19 linkage groups were localized to 10 pairs of chromosome [16]. In the present study, 30 of the 38 BAC clones produced specific FISH signals on one pair of chromosomes. Twenty-six BAC clones need to be added *C_0_t*-1 DNA to block the background signals and produce specific signal, which suggested that a large proportion of these BAC clones consisted of repetitive DNA. Using 30 markers-anchored BAC clones, 18 of 19 linkage groups were assigned by FISH to 17 pairs of chromosomes by karyotyping analysis. However, because of the similar morphology and high number of *C. farreri* chromosomes, the correspondence between chromosomes and linkage groups may not be very accurate only based on karyotyping analysis. Therefore, two-color FISH was performed, which confirmed six linkage groups could be assigned to different chromosomes. But since 70% of the probes could only produce unique signals with *C_0_t*-1 DNA and these probes could not produce specific signals in two-color FISH, it is still uncertain and needs to be further verified the correspondence between some other linkage groups and chromosomes. In addition, two linkage groups (LG16 and LG18) which only have several markers were integrated to the long linkage groups (LG6 and LG 8) separately. The order of markers in 8 linkage groups was consistent with the distribution of BAC clones on chromosomes. The orientation of 3 linkage groups (LG1, LG3, and LG10) was revised by the position of the BAC clones. Four linkage groups were separated because the two BAC clones from the same linkage group were localized on two different chromosomes. In summary, the molecular genetic map could not only integrate the linkage groups to the chromosomes, but also revise the marker order and orientation of linkage groups.

The recombination rate is always variable among different chromosomal region. In general, genetic distance is more than physical distance when molecular markers are in the distal region of a chromosome. This phenomenon has been reported in many species with large genomes and high repetitive DNA sequences, such as wheat whose 99% of the recombination occurs in the distal 60% of the arm [40]. In sorghum, the relationship between genetic distance and physical distance was demonstrated by probing a 14-BAC probe cocktail, which suggested only ∼1.7 of the 242.9 map units were found to span ∼60% of the physical length of chromosome 1 [41]. In pacific oyster, the differences in recombination and the order of markers on linkage groups among different family have been observed, which might indicate biological variation in recombination rate [42]. In our research, the relationship between genetic distance and physical distance was found to be inconsistent in LG5, Lg11 and LG12. For instance, the two markers CFKD096 and CFKD091 in LG12 have a genetic distance of 37.55%. Nevertheless, the physical distance between these markers is only 8.33% of the total length of chromosome 11. This inconsistent relationship between genetic and physical distances may also be affected by the relatively low density of markers on the current genetic map. In addition, our FISH mapping results also suggested that the genetic distance of different chromosomes does not always corresponding to their physical distance, indicating that the current genetic map may not fully cover the whole genome. For example, the two markers CFFD144 and CFAD018 in LG5 have a genetic distance of 84.52%. Nevertheless, the physical distance between these markers is only 36.75% of the total length of chromosome 18.

The integrated molecular genetic maps will provide a starting point for genome assembly of Zhikong scallop, *C. farreri*. This could greatly facilitate molecular genetic studies which will be helpful for chromosomal localization of genes and identification of major genes associated with economically important traits.

## Supporting Information

Figure S1 BAC-FISH Karyotype of LG1, 2, 3, and 4.
(TIF)

Figure S2 BAC-FISH Karyotype of LG5, 6, 7, 8 and 9.
(TIF)

Figure S3 BAC-FISH Karyotype of LG10, 11, 12, 13, 14, and 15.
(TIF)

Figure S4 BAC-FISH Karyotype of LG16, 17, 18, and 19.
(TIF)

Table S1 Information of 38 microsatellite loci and BAC library screening results.
(DOC)
