# Correction: An Integrated Genetic and Cytogenetic Map for Zhikong Scallop, *Chlamys farreri*, Based on Microsatellite Markers

**DOI:** 10.1371/journal.pone.0103167

**Published:** 2014-07-14

**Authors:** The PLOS ONE Staff

In the Funding section, the grant number from the funder National Natural Science Foundation of China is listed incorrectly. The correct grant number is: 31270047.

## References

[1] Feng L, Hu L, Fu X, Liao H, Li X, et al (2014) An Integrated Genetic and Cytogenetic Map for Zhikong Scallop, *Chlamys farreri*, Based on Microsatellite Markers. PLoS ONE 9(4): e92567 doi:10.1371/journal.pone.0092567 24705086 10.1371/journal.pone.0092567 PMC3976258

