# Supplementary figures and images for: An Integrated Genetic and Cytogenetic Map for Zhikong Scallop, Chlamys farreri, Based on Microsatellite Markers
Source: PLoS One. 2014 Apr 4;9(4):e92567. doi: 10.1371/journal.pone.0092567 (PMC3976258; doi:10.1371/journal.pone.0092567)

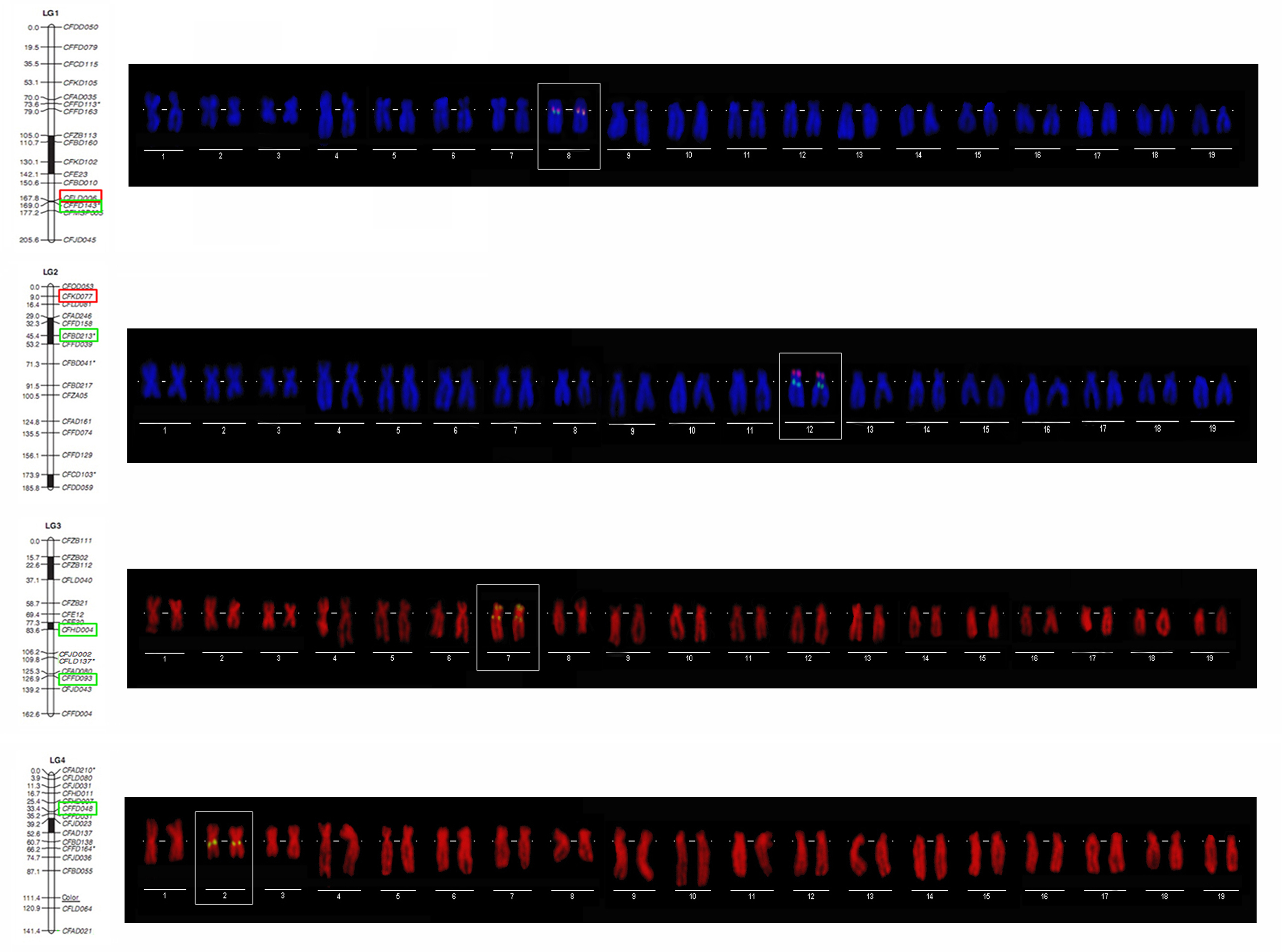

Supplement: Figure S1 — BAC-FISH Karyotype of LG1, 2, 3, and 4. (TIF) [file pone.0092567.s001.tif]

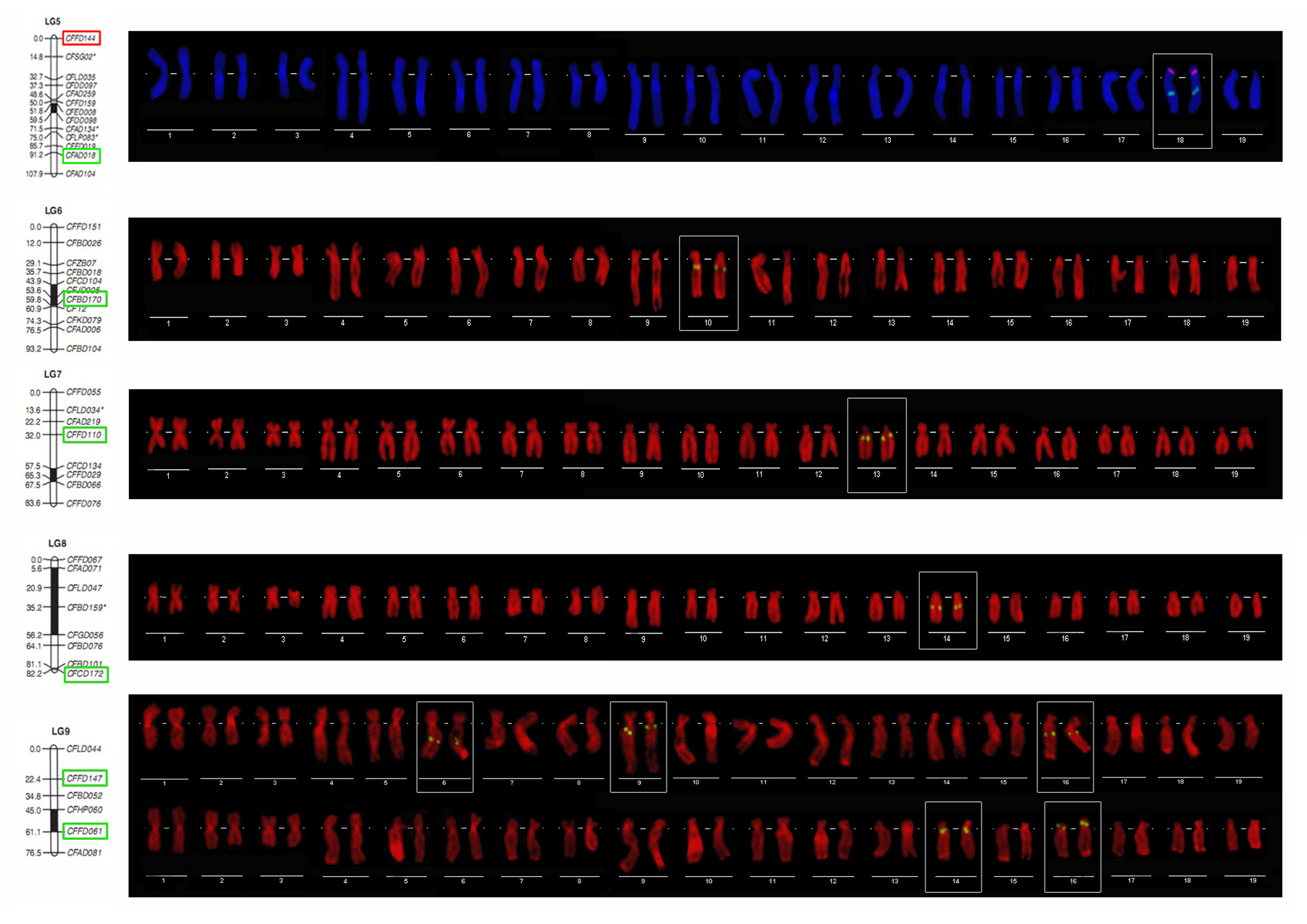

Supplement: Figure S2 — BAC-FISH Karyotype of LG5, 6, 7, 8 and 9. (TIF) [file pone.0092567.s002.tif]

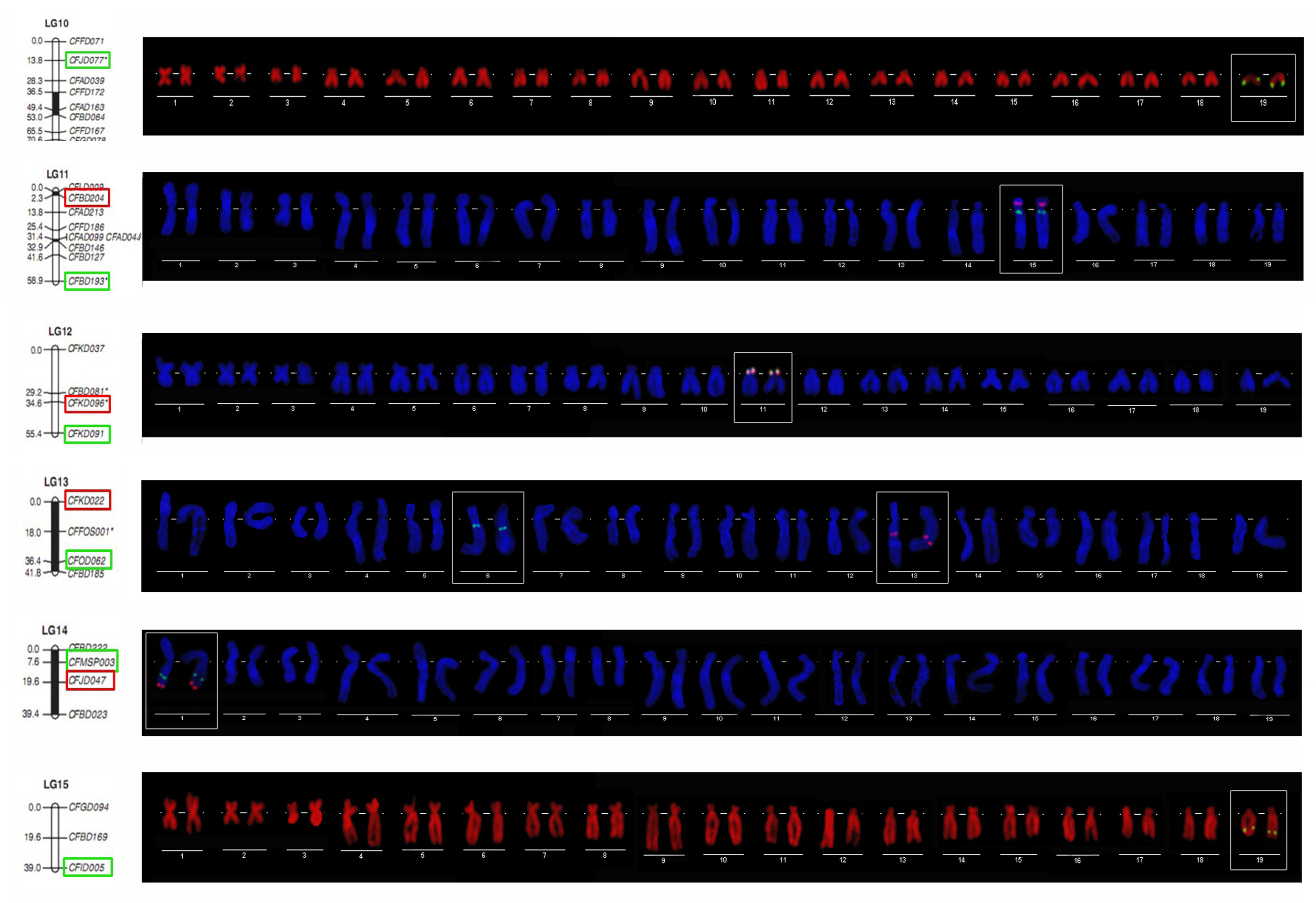

Supplement: Figure S3 — BAC-FISH Karyotype of LG10, 11, 12, 13, 14, and 15. (TIF) [file pone.0092567.s003.tif]

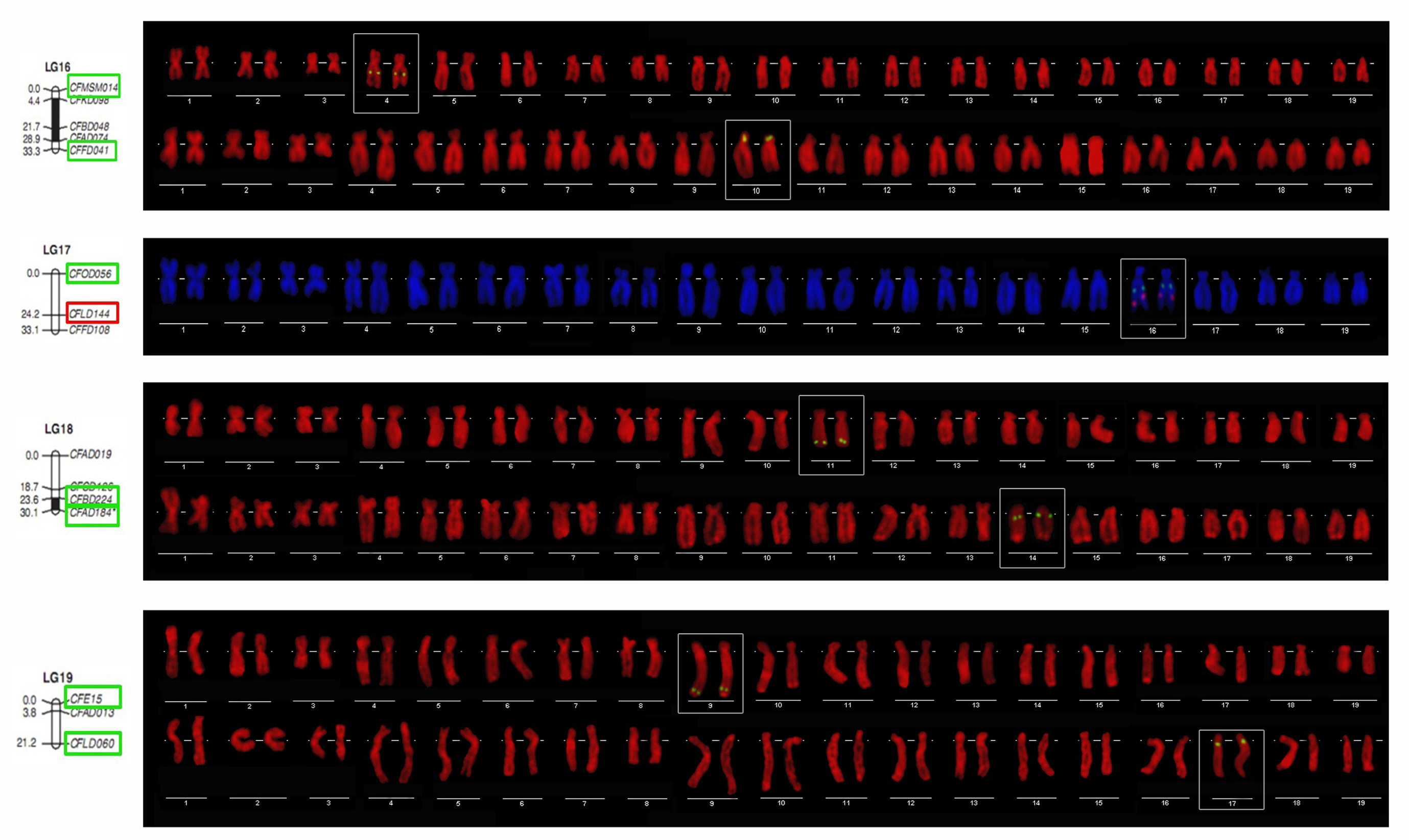

Supplement: Figure S4 — BAC-FISH Karyotype of LG16, 17, 18, and 19. (TIF) [file pone.0092567.s004.tif]
