# Supplementary material for: An Integrated Genetic and Cytogenetic Map for Zhikong Scallop, Chlamys farreri, Based on Microsatellite Markers
Source: PLoS One. 2014 Apr 4;9(4):e92567. doi: 10.1371/journal.pone.0092567 (PMC3976258; doi:10.1371/journal.pone.0092567)
Supplement: Table S1 — Information of 38 microsatellite loci and BAC library screening results. (DOC) [file pone.0092567.s005.doc]

**Table S1. Information of 38 microsatellite loci and BAC library screening results.**

| **Linkage Group** | **Locus name** | **Primer sequences (5’-3’)** | **Repeats** | **BAC code** |
| --- | --- | --- | --- | --- |
| **LG1** | CFLD006  (EF148974) | F:AGGGTTTCAAATGATACGGG  R: GGATGGCAAGATTAAATGAGC | (GA)21 | BH1304E11 |
| **LG1** | CFFD143  (EF148943) | F:CGCCAACCTTGCAGTATCTG  R: TTCTTTCCCTCTTCTGTCCC | (GA)3CA(GA)3  GGAA(GA)11 | BH1162H2 |
| **LG2** | CFBD213  (EF148908) | F: CTGGCTGTGCTGGATGGAC  R:CAAAGAATTCAAATGGATTGGGC | (CT)4…(CT)4  …(CT)7…(CT)5 | BH783B4 |
| **LG2** | CFKD077  (EF552140) | F:CATATAGGGCAGGAATTGCG  R:GTCAAGTGATGATGGACATAGC | (AG)27 | BH793B11 |
| **LG3** | CFHD004  (EF552131) | F: GACACCGTCTCGCAATCTG  R:GCAACGTAATAAGGCACAAC | (CT)36 | BH799B12 |
| **LG3** | CFFD093  (EF392892) | F: GAATTACACATCATAAGATGGC  R:AGAAGGGAATAGTGGCAGGT | (GA)26 | BH1049A9 |
| **LG4** | CFFD048  (EF392889) | F: ACATCGTGTTCCCCTGACC  R:TGACCAAACCACGGACTTAG | (GA)17 | BB311A9 |
| **LG4** | CFAD021  (EF148858) | F:CAGTTACCTGTTATCTACCTCCC  R: AATCCTCGATCTGCCTCAC | (AG)16…(AG)8…(AG)12…(AG)10 | BB322D4 |
| **LG5** | CFFD144  (EF148944) | F: TTGTCTCCAAACAGAAATCC  R:CCTCTGTTCATTTTCATCAAGC | (AG)5AA(AG)2AA(AG)5GG(AG)22GG(AG)6 | BB105A1 |
| **LG5** | CFAD018  (EF148856) | F:GTTTTACGCCTTATTAACAGC  R:GTCGGTATCAGATCACTATCAC | (AAGCGTGCTGGCATGTGTGTAGTGT)6 | BB86D4 |
| **LG6** | CFBD170  (EF148903) | F:GACAACAAAGTGCCACCTAAGC  R:AAAACACCGTTCCCCCTTCAG | (GA)16 | BH1285H8 |
| **LG6** | CFCD104  (EF148912) | F:GATTTTTCCAACGTCAGACTC  R: ATCTCCTGTCCATTCATCTC | (CAT)4…(CA)3…(CA)9CT(AC)7…(GA)4…(GA)5 | BB24H11 |
| **LG7** | CFFD110  (EF148939) | F: ACACCCTTCCTTACCTACAG  R: GTCAGATGTTCTTATCCCTA | (CT)33 | BH1291D12 |
| **LG7** | CFLD034  (EF148976) | F:CTAAATCAACACAACTAAGCC  R:GAAGATGAAGTCTAACTGCC | (TC)16…(CT)7 | BH1060G11 |
| **LG8** | CFCD172  (EF148917) | F:GAGCCCTATAAATTCCCCCTG  R: GATGGATTTTTCCGCCTACG | (CA)13CC(CA)12…(CA)10 | BH966F2 |
| **LG8** | CFLD047  (EF148980) | F: CATCACAACCTTCCGAACGCAC  R: GCCAACCACAATGTCAAATC | (GA)20 | BB138H4 |
| **LG9** | CFFD147  (EF148945) | F:CATTTACTACCCACGACCAACC  R:TCTCCGGGCCTTGAGATATG | (AG)15 | BB224E11 |
| **LG9** | CFFD061  (EF148930) | F:GATTTAAGGTGACAATTAGTGG  R:ACGCTAAACCTAAAACAGAAG | (GA)39 | BB98C5 |
| **LG10** | CFJD077  (EF392900) | F: GACCCCAATCCACATCATC  R:GCAAAAGATGGCCTTTTTGAAAG | (CT)2 | BB39F11 |
| **LG10** | CFFD167  (EF148954) | F:GAATACATAGTGTTTCACAGT  R:CCGTCTCGTCAAAAGATTAC | (AG)15 | BB27C6 |
| **LG11** | CFBD204  (EF148907) | F:TGACCTCTGACTCCACTCG  R:CACACCAGGAGCAAATAAAC | (CA)16 | BB75B6 |
| **LG11** | CFBD193  (EF148906) | F:GGATGTAACCGATAAATATGGC  R:TGAAGGCGATGTTGGTGCTC | (AG)10AA(AG)7 | BH1308E3 |
| **LG12** | CFKD091 | F:GACAATACCGTGTACAGTATG  R:CTATCCATTCTCCCTTCCTTG | (AG)21 | BH885H2 |
| **LG12** | CFKD096  (EF148970) | F: CTCTAACATTCCGAGACATC  R: CGGTATTTTACATTCCAAGG | (TC)10TG(TC)5TG(TC)13 | BB224B4 |
| **LG13** | CFOD062  (EF552148) | F:GCTGGAAAGCAAACTCATGTAAGG  R:GAGATGAACCAGAAGTAAGTAAAG | (AG)7…(AG)9(AC)5(AG)10 | BB233G7 |
| **LG13** | CFKD022  (EF148964) | F:CTAGCCTACACGGTATTCAAC  R:TCGTAGGAAACGCACGCAGT | (CT)22 | BB312B11 |
| **LG14** | CFMSP003  (AY682109) | F:CGACTCTGCCCCTAGTGTCTTC  R:AACAAGGGTACTTCACGGTCGG | (AGC)9 | BH984B5 |
| **LG14** | CFJD047  (EF392899) | F:GTTTTATGTAAGAAACGTAGTC  R:GTATTGACCCTACTAACCC | (GA)33 | BB105B2 |
| **LG15** | CFID005  (EF552134) | F:CACACAAATGGATTGGGCAC  R:GACCGAAATCCACATCATCTA | (GA)31 | BB235A11 |
| **LG15** | CFBD169  (EF148902) | F:TGATTGGCGAAGCGTTGTAG  R:ACATGACGATACTTGGTGGAAG | (GA)21 | BH377G2 |
| **LG16** | CFMSM014  (DQ104705) | F:CATCTGATATGGCAGCTGATAC  R:GAACTAACGAGGAGACAACTG | (AG)10…(AC)4 | BH565D6 |
| **LG16** | CFFD041  (EF017165) | F:CATATCTACACTGTCACCCTC  R:GCTTATGCAGGACTTGGAAT | (AG)16 | BB69B10 |
| **LG17** | CFLD144  (EF148992) | F:GCTGAAAGAATGGAATTGTGC  R:TGTCAAATCGGTGGTATGTG | (CT)17…(CT)21 | BH368F12 |
| **LG17** | CFOD056  (EF552147) | F:TGATGAAGCAAAGTCAAGAG  R:GAGTCTGAAAGTTCGTAAGCC | (CT)5GT(CT)5GT  (CT)4…(CT)16 | BH1261C3 |
| **LG18** | CFAD184  (EF148871) | F: ACATTATTTACGCTGTATGGAC  R:TGAAAGAATGGAATAGTGCC | (GA)14(G)11(AG)10 | BH986B2 |
| **LG18** | CFBD224  (EF392882) | F:GAGAAAATTCTGAAAACGCTC  R:AGGCATCGGTCTACGGCTAA | (CA)7…(CA)3CC(CA)7 | BH1003G1 |
| **LG19** | CFLD060  (EF148904) | F:GCCTTTGACTGACTAACATTC  R: AGTGCTTGCTGATTTTTCGC | (AG)14(ACAG)3(AG)6 | BB239A6 |
| **LG19** | CFE15  (DT718945) | F: CTCGCCCTCCTTTTACACAC  R:GTCCTACAGGTAACTTTGACTATC | (TATAC)5 | BH431C4 |
